# The optimal exercise for improving inhibitory control in children and adolescents with NDDs: a systematic review and network meta-analysis

**DOI:** 10.3389/fpsyt.2026.1818708

**Published:** 2026-05-20

**Authors:** Tianyang Zhang, Fanzhi Zeng, Feng Li, Bowen Niu

**Affiliations:** 1 Ice and Snow College, Zhangjiakou University, Zhangjiakou, Hebei, China; 2 Zhangjiakou No.9 Middle School, Zhangjiakou, Hebei, China; 3 Langfang Yanjing Vocational and Technical College, Langfang, Hebei, China

**Keywords:** children and adolescents, exercise type, inhibitory control, network meta-analysis, neurodevelopmental disorders

## Abstract

**Objective:**

Inhibitory control refers to an individual’s ability to overcome distractions. This deficit is prevalent among children and adolescents with neurodevelopmental disorders (NDDs), whilst pharmacological treatments carry significant side effects. This study employed a network meta-analysis to compare the effects of different types of exercise on inhibitory control in children and adolescents with NDDs, to provide evidence-based guidance for the selection of optimal clinical interventions.

**Methods:**

Five electronic databases were searched from inception to March 1, 2025. Randomized controlled trials were selected, the Cochrane tool was used to assess the risk of bias, the standardized mean difference (SMD) was adopted as the effect size, and Stata 17.0 was used to conduct the network meta-analysis. The intervention effects of each exercise modality were ranked through SUCRA analysis.

**Results:**

This study included a total of 45 studies involving 2, 358 participants (mean age 9.0 ± 2.6 years). The rehabilitation interventions identified and included in this study comprised physical and mind-body exercises, cycling, ball sports, and others. The network meta-analysis showed that aerobic exercise (SMD = 0.71, 95% CI 0.15–1.26, P < 0.05) and mind-body exercise (SMD = 0.81, 95% CI 0.19–1.42, P < 0.05) were significantly superior to the conventional control group. SUCRA probability ranking results indicated that mind-body exercise (SUCRA = 87.1) may be the optimal exercise modality for improving inhibitory control in children and adolescents with neurodevelopmental disorders.

**Conclusion:**

This study suggests that mind-body exercise may be the optimal exercise type for improving inhibitory control function in children with neurodevelopmental disorders and may provide guidance for clinical interventions. However, due to potential influences from factors such as gender, age, and individual differences, this conclusion should be interpreted with caution.

**Systematic Review Registration:**

https://www.crd.york.ac.uk/PROSPERO/view/CRD420251125648, identifier CRD420251125648.

## 1 Introduction

Neurodevelopmental disorders (NDDs) are a group of conditions that emerge during early childhood and adolescence, affecting the maturation and functional networks of the brain. They primarily include attention-deficit/hyperactivity disorder (ADHD), autism spectrum disorder (ASD), mild intellectual disability (MID), learning disabilities (LD), and developmental coordination disorder (DCD), among others (1, 2). Among the various functional deficits associated with NDDs, inhibitory control is often considered a core deficit, characterized by impaired ability to inhibit inappropriate or impulsive responses, and has been a focal point of NDD research (3, 4). Its occurrence is closely associated with neurophysiological abnormalities and damage to motor control pathways. From a neurophysiological perspective, the core issue underlying the reduced inhibitory control in patients with NDDs is a dysfunction of the prefrontal-striatal-thalamic circuit (5). As the central hub for inhibitory control, the prefrontal cortex exhibits reduced grey matter volume, weakened neuronal connections and neurotransmitter abnormalities, all of which impair its regulatory function (6). From the perspective of motor control mechanisms, impaired motor inhibitory pathways in patients with NDD prevent the effective suppression of excessive motor responses (7). Furthermore, reduced conduction efficiency in the corticospinal tract leads to hyperexcitability of the primary motor cortex, which may further exacerbate motor inhibition deficits (8).While there is extensive research on individual NDDs, few studies have systematically compared the effects of different types of exercise on inhibitory control across various NDDs. Therefore, the purpose of this study is to explore the effects of different types of exercise on inhibitory control in children and adolescents with NDDs and to identify the optimal intervention type.

According to existing global epidemiological surveys, the overall prevalence of NDDs ranges from 4.70% to 88.50%, and it is believed that single NDDs rarely occur, but rather manifest as overlapping between different NDDs and with other mental disorders. Therefore, NDDs should be considered as a whole, and their comorbidity patterns should be systematically explored (9). This condition places a significant burden on the healthcare system, as NDDs lead to a continuous increase in outpatient visits, medication prescriptions, and rehabilitation service demands (10). Some studies predict that healthcare expenditures related to autism in the United States may reach as high as $461 billion by 2025 (11). On the other hand, this condition also imposes a substantial burden on families, with parents spending an average of 26.7 additional hours per week on treatment, which may lead to a decline in family quality of life (12). Additionally, this condition may result in adolescents having lower academic performance compared to their peers, increased dropout rates, and reduced employment rates and income levels in adulthood (13).

Currently, interventions for such symptoms primarily rely on central nervous system stimulants (e.g., methylphenidate, amphetamine) and non-stimulants (e.g., atomoxetine, guanfacine). While these can improve core symptoms in the short term, they are associated with side effects such as addiction risk, tolerance, appetite suppression, sleep disorders, and mood fluctuations. Long-term use may also lead to dose escalation and drug dependence (14). Therefore, non-pharmacological interventions have become a focal point of academic research. In recent years, exercise therapy has emerged as a new direction in intervention studies due to its advantages of zero addiction potential and low cost. Neurobiological studies have shown that exercise therapy exerts positive effects on the brain through neurogenesis, neural adaptation, and neuroprotection, thereby improving participants’ inhibitory control (15).Specifically, with regard to neurogenesis, exercise upregulates the expression of brain-derived neurotrophic factor and vascular endothelial growth factor, promoting the proliferation and differentiation of neural progenitor cells in the hippocampus and prefrontal cortex, and enhancing the structural plasticity of executive control circuits (16). In terms of neural adaptation, exercise remodels dopamine and noradrenaline function, optimizes neural projections between the midbrain and prefrontal cortex, and enhances the efficiency of response inhibition, as evidenced by shorter stop-signal reaction times (17). In terms of neuroprotection, exercise inhibits excessive activation of microglia, mitigates oxidative damage in prefrontal and striatal circuits, and maintains the functional stability of inhibitory control neural networks (18). Further research has shown that acute aerobic exercise can increase the amplitude of the P3 component in event-related potentials, indicating that response conflict and stimulus classification speed are generally enhanced, further demonstrating the effectiveness of exercise in improving participants’ inhibitory control (19). In inhibition control tasks, the increased amplitude of the P3 waveform elicited by No-Go stimuli reflects the need to suppress dominant responses; this increase in P3 amplitude also indicates improved working memory integration efficiency, which facilitates rapid decision-making and thereby enhances the efficiency of interference suppression (20).

Although some studies have shown through meta-analysis that chronic exercise may be most effective in improving inhibitory control in such populations (21), traditional meta-analysis methods for exploring the effects of exercise interventions can only combine direct comparative evidence when faced with multiple intervention protocols, making it difficult to systematically integrate indirect information. This prevents a comprehensive comparison of the relative efficacy of different interventions and the establishment of a clear ranking of their advantages and disadvantages. Network meta-analysis can utilize both direct and indirect evidence to overcome the limitations of traditional meta-analysis, enabling a comprehensive quantification and ranking of all intervention measures, thereby providing more complete and precise evidence for clinical decision-making (22). Therefore, this study employed network meta-analysis to comprehensively evaluate the relative effects of different types of exercise interventions on inhibitory control in children and adolescents with neurodevelopmental disorders, aiming to provide evidence-based guidance for selecting the optimal exercise type in clinical practice.

## 2 Methods

This systematic review and network meta-analysis was carried out in accordance with the Cochrane Intervention Systematic Review Manual, and registration was completed on the PRISMA (Preferred Reporting Items for Systematic Review and Meta-Analysis) registration website before the launch of the network meta-analysis. The registration number is CRD420251125648 (23).

### 2.1 Literature search strategy

This study retrieved randomized controlled trials from five databases (Web of Science, PubMed, EBSCO, Embase, and Cochrane) from the establishment of the databases to March 2025. The following combination of search terms was used: (1) aerobic exercise, resistance training, strength training, endurance training, ball sports, racket sports, table tennis, badminton, basketball, soccer, martial arts, taekwondo, karate, yoga, mind-body exercise, exergaming, coordination training, balance training, physical activity intervention, task-oriented training, neuromotor task training; (2) children, adolescents, youth, teenagers, school-age, pupils, primary school students, middle school students, high school students, 6 years, 7 years, 8 years, 9 years, 10 years, 11 years, 12 years, 13 years, 14 years, 15 years, 16 years, 17 years; (3) neurodevelopmental disorders, developmental coordination disorder (DCD), attention deficit hyperactivity disorder (ADHD), ADHD, autism spectrum disorder, ASD, mild intellectual disability, borderline intellectual functioning, learning disabilities, specific learning disorder, MID, ED, BIF, LD, SLD. The Boolean operator “AND” was used to connect the three groups of terms, linking related thematic literature to ensure the inclusion of key elements in the search and guaranteeing the comprehensiveness of the retrieval. Detailed information on the search strategy is provided in **Table 1**.

**Table 1:** Complete search strategy for databases.

Web of science search strategy		
Step	Field	Search string
1	TS=	("aerobic exercise" OR "endurance training" OR "resistance training" OR "strength training"OR "ball sports" OR "racket sports" OR "table tennis" OR badminton OR basketball OR soccer OR "martial arts" OR taekwondo OR karate OR yoga OR "mind-body exercise" OR exergaming OR "coordination training" OR "balance training" OR "physical activity intervention" OR "task-oriented training" OR "neuromotor task training")
2	TS=	(child* OR adolescen* OR youth* OR teenager* OR "school-age" OR pupil* OR "primary school student*" OR "middle school student*" OR "high school student" OR "6 years" OR "7 years" OR "8 years" OR "9 years" OR "10 years" OR "11 years" OR "12 years" OR "13 years" OR "14 years" OR "15 years" OR "16 years" OR "17 years")
3	TS=	("neurodevelopmental disorders" OR "developmental coordination disorder" OR DCD OR "attention deficit hyperactivity disorder" OR ADHD OR "autism spectrum disorder" OR ASD OR "mild intellectual disability" OR "borderline intellectual functioning" OR "learning disabilities" OR "specific learning disorder" OR MID OR ED OR LD OR SLD)
4	Combine	#1 AND #2 AND #3

PubMed search strategy		
Step	Field & tags	Search string
1	(Title/Abstract[Title/Abstract])	("aerobic exercise"[Title/Abstract] OR "endurance training"[Title/Abstract] OR "resistance training"[Title/Abstract] OR "strength training"[Title/Abstract] OR"ball sports"[Title/Abstract] OR "racket sports"[Title/Abstract] OR "table tennis"[Title/Abstract] OR badminton[Title/Abstract] OR basketball[Title/Abstract] OR soccer[Title/Abstract] OR "martial arts"[Title/Abstract] OR taekwondo[Title/Abstract] OR karate[Title/Abstract] OR yoga[Title/Abstract] OR "mind-body exercise"[Title/Abstract] OR exergaming[Title/Abstract] OR "coordination training"[Title/Abstract] OR "balance training"[Title/Abstract] OR "physical activity intervention"[Title/Abstract] OR "task-oriented training"[Title/Abstract] OR "neuromotor task training"[Title/Abstract])
2	(Title/Abstract[Title/Abstract])	(child*[Title/Abstract] OR adolescen*[Title/Abstract] OR youth*[Title/Abstract] OR teenager*[Title/Abstract] OR "school-age"[Title/Abstract] OR pupil*[Title/Abstract] OR "primary school student*"[Title/Abstract] OR "middle school student*"[Title/Abstract] OR "high school student*" [Title/Abstract] OR "6 years"[Title/Abstract] OR "7 years"[Title/Abstract] OR "8 years"[Title/Abstract] OR "9 years"[Title/Abstract] OR "10 years"[Title/Abstract] OR "11 years"[Title/Abstract] OR "12 years"[Title/Abstract] OR "13 years"[Title/Abstract] OR "14 years"[Title/Abstract] OR "15 years"[Title/Abstract] OR "16 years"[Title/Abstract] OR "17 years"[Title/Abstract])
3	(Title/Abstract[Title/Abstract]) OR (MeSH Terms)	("neurodevelopmental disorders"[Title/Abstract] OR "developmental coordination disorder"[Title/Abstract] OR DCD[Title/Abstract] OR "attention deficit hyperactivity disorder"[Title/Abstract] OR ADHD[Title/Abstract] OR "autism spectrum disorder"[Title/Abstract] OR ASD[Title/Abstract] OR "mild intellectual disability"[Title/Abstract] OR "borderline intellectual functioning"[Title/Abstract] OR "learning disabilities"[Title/Abstract] OR "specific learning disorder"[Title/Abstract] OR MID[Title/Abstract] OR ED[Title/Abstract] OR LD[Title/Abstract] OR SLD[Title/Abstract] OR "Neurodevelopmental Disorders"[MeSH] OR "Developmental Disabilities"[MeSH] OR "Attention Deficit and Disruptive Behavior Disorders"[MeSH] OR "Autism Spectrum Disorder"[MeSH] OR "Learning Disorders"[MeSH])
4	Combine	#1 AND #2 AND #3

EBSCO search strategy		
Step	Field	Search string
1	KW	("aerobic exercise" OR "endurance training" OR "resistance training" OR "strength training" OR "ball sports" OR "racket sports" OR "table tennis" OR badminton OR basketball OR soccer OR "martial arts" OR taekwondo OR karate OR yoga OR "mind-body exercise" OR exergaming OR "coordination training" OR "balance training" OR "physical activity intervention" OR "task-oriented training" OR "neuromotor task training")
2	KW	(child* OR adolescen* OR youth* OR teenager* OR "school-age" OR pupil* OR "primary school student*" OR "middle school student*" OR "high school student*" OR "6 years" OR "7 years" OR "8 years" OR "9 years" OR "10 years" OR "11 years" OR "12 years" OR "13 years" OR "14 years" OR "15 years" OR "16 years" OR "17 years")
3	KW	("neurodevelopmental disorders" OR "developmental coordination disorder" OR DCD OR "attention deficit hyperactivity disorder" OR ADHD OR "autism spectrum disorder" OR ASD OR "mild intellectual disability" OR "borderline intellectual functioning" OR "learning disabilities" OR "specific learning disorder" OR MID OR ED OR LD OR SLD)
4	Combine	S1 AND S2 AND S3

Embase search strategy		
Step	Field & tags	Search string
1	Title, Abstract, Keywords	('aerobic exercise' OR 'endurance training' OR 'resistance training' OR 'strength training' OR 'ball sports' OR 'racket sports' OR 'table tennis' OR badminton OR basketball OR soccer OR 'martial arts' OR taekwondo OR karate OR yoga OR 'mind-body exercise' OR exergaming OR 'coordination training' OR 'balance training' OR 'physical activity intervention' OR 'task-oriented training' OR 'neuromotor task training')
2	Title, Abstract, Keywords	(child* OR adolescen* OR youth* OR teenager* OR 'school-age' OR pupil* OR 'primary school student*' OR 'middle school student*' OR 'high school student*' OR '6 years' OR '7 years' OR '8 years' OR '9 years' OR '10 years' OR '11 years' OR '12 years' OR '13 years' OR '14 years' OR '15 years' OR '16 years' OR '17 years')
3	Title, Abstract, Keywords OR Emtree Terms	('neurodevelopmental disorders' OR 'developmental coordination disorder'/exp OR dcd OR 'attention deficit hyperactivity disorder'/exp OR adhd OR 'autism spectrum disorder'/exp OR asd OR 'mild intellectual disability' OR 'borderline intellectual functioning' OR 'learning disabilities' OR 'specific learning disorder' OR mid OR ed OR ld OR sld)
4	Combine	#1 AND #2 AND #3

Cochrane search strategy		
Step	Field	Search string
1	Title Abstract Keywords	("aerobic exercise" OR "endurance training" OR "resistance training" OR "strength training" OR "ball sports" OR "racket sports" OR "table tennis" OR badminton OR basketball OR soccer OR "martial arts" OR taekwondo OR karate OR yoga OR "mind-body exercise" OR exergaming OR "coordination training" OR "balance training" OR "physical activity intervention" OR "task-oriented training" OR "neuromotor task training")
2	Title Abstract Keywords	(child* OR adolescen* OR youth* OR teenager* OR "school-age" OR pupil* OR "primary school student*" OR "middle school student*" OR "high school student*" OR "6 years" OR "7 years" OR "8 years" OR "9 years" OR "10 years" OR "11 years" OR "12 years" OR "13 years" OR "14 years" OR "15 years" OR "16 years" OR "17 years")
3	Title Abstract Keywords OR MeSH	("neurodevelopmental disorders" OR "developmental coordination disorder" OR DCD OR "attention deficit hyperactivity disorder" OR ADHD OR "autism spectrum disorder" OR ASD OR "mild intellectual disability" OR "borderline intellectual functioning" OR "learning disabilities" OR "specific learning disorder" OR MID OR ED OR LD OR SLD)
4	Combine	#1 AND #2 AND #3

### 2.2 Literature inclusion and exclusion criteria

Inclusion and exclusion criteria were established according to the PICOS principle. (1) P: Children and adolescents under the age of 18 who have been diagnosed with any neurodevelopmental disorder (DCD, ADHD, ASD, ED, mild intellectual disability, borderline intellectual functioning, learning disabilities, specific learning disorder). (2) I: Any motor intervention (aerobic exercise, ball sports, racket sports, martial arts, yoga, exergaming, coordination/balance training, task-oriented training, etc.), implemented alone or in combination, with no restrictions on duration, frequency, or intensity. (3) C: Conventional treatment or non-exercise control (conventional rehabilitation, standard education, routine care). (4) O: At least one validated inhibitory control assessment tool (Go/No-Go, Stroop, Flanker, Stop-Signal task, etc.) was used, with pre- and post-intervention data or change values reported. (5) S: Published randomized controlled trials (RCTs), language restricted to English, publication status unrestricted. Two researchers independently screened the studies, deduplicated using EndNote, and any discrepancies were arbitrated by a third researcher. Exclusion criteria were as follows: (1) Participants aged over 18 years or diagnosed with non-neurodevelopmental disorders such as cerebral palsy, epilepsy, or mood disorders; (2) Interventions primarily involving medication, diet, transcranial stimulation, or psychological interventions, or where the exercise component accounted for less than 50% of the total intervention duration; (3) Studies that did not report data specifically related to inhibitory control, only providing total executive function scores; (4) Non-randomized designs, single-arm trials, case reports, reviews, conference abstracts without full text, duplicate publications, or studies where data could not be extracted.

### 2.3 Data extraction

Both researchers (TYZ and BWN) were trained in evidence-based methodology. They independently extracted data using pre-designed standardized tables. First, the information in the included literature was cross-checked, and then was extracted the following key information from each article, including: the name of the first author, the specific publication year, the country in which the study was conducted, the sample size of children and adolescents participating in the study (including n values for the intervention group and the control group), the specific type of intervention measures implemented, and the main outcome indicators used to evaluate the intervention effect, including the name and measurement values of inhibitory control tasks. If any differences occurred during the extraction process, a third researcher (FZZ) reviewed the original literature and discussed it with the first two researchers until an agreement was reached, to ensure the integrity and accuracy of the data. If data were missing, the corresponding author was contacted directly via email to request additional information.

### 2.4 Risk of bias evaluation of included studies

Two researchers independently used the Cochrane RoB 2.0 tool to evaluate the risk of bias in each randomized controlled trial (RCT) across the following seven domains: random sequence generation, allocation concealment, blinding of participants and personnel, blinding of outcome assessment, completeness of outcome data, selective reporting, and other potential biases. After each domain was evaluated, the overall risk of bias for each study was classified into three categories: low risk, high risk, or some concerns (unclear risk). If disagreements occurred between the two reviewers, the original text was reviewed and discussed. If necessary, a third senior researcher was consulted to mediate until a consensus was finally reached. Finally, a summary graph of the risk of bias was generated.

### 2.5 Statistical methods

A consistency test was conducted on the global level. The test results showed that the p-value for the global consistency test was 0.265, which is greater than 0.05, indicating good global consistency. Further tests were conducted on the consistency of each closed loop. The closer the inconsistency factor is to 0, the better the consistency. The test results showed that the lower limit of the inconsistency factor included 0, indicating that the consistency of the loops is good, suggesting that there is no significant inconsistency between direct and indirect comparison results. Additionally, all analyses were conducted using Stata 17.0 software. Given that the outcome of inhibitory control is continuous and the assessment tools and measurement units used in each study vary, the standardized mean difference (SMD) was adopted as the effect size, and its 95% confidence interval was calculated to precisely pool the effects. SUCRA (Sum of Cumulative Rankings Area) was further utilised to quantify the relative efficacy of each exercise intervention. This analysis was based on the effect sizes (SMD) derived from the network meta-analysis; subsequently, the mean ± standard deviation following intervention was extracted for the included outcome measures, and the cumulative probability area for each intervention across all possible rankings was calculated. SUCRA values range from 0 to 100, with higher values indicating a greater likelihood of being the optimal treatment option. In this study, some RCTs included multiple active intervention arms sharing the same control group. To avoid overestimating precision due to double-counting of control group participants, a node-splitting strategy was employed, pairing each intervention arm with a split control group to form independent comparison pairs. All multi-arm studies were incorporated into the evidence network following this processing to ensure the statistical validity of the network meta-analysis.

### 2.6 Evidence certainty assessment

The GRADE criteria were applied to assess the quality of evidence regarding inhibitory control in children with neurodevelopmental disorders (see **Table 2**). The results indicated that the quality of evidence for various motor interventions producing significant improvements in inhibitory control in this population was moderate.

**Table 2:** Grading of recommendations assessment, development and evaluation.

Outcomes	Presence of downgrading item of GRADE					Level of certainty of evidence
	Risk Of bias	Inconsistency	Indirectness	Imprecision	Publication bias	
Inhibitory control	Yes	No	No	No	No	II (Moderate) (1)

(1) Risk of bias: Risk of bias exists in the studies included in the meta-analysis regarding random sequence generation, allocation concealment, or blinding of participants and assessors; (2) Inconsistency: Point estimates are concentrated, confidence intervals may overlap, and the results of heterogeneity tests are not statistically significant; (3) Indirectness: The interventions and outcomes investigated in the meta-analysis are not directly related; (4) Imprecision: The total sample size of all individual studies in the meta-analysis is less than 500, and the 95% CI of the effect size is relatively wide; (5) Publication bias: Publication bias is considered present if authors only searched Chinese databases or only one database.

## 3 Results

### 3.1 Results of literature search

**Figure 1:**
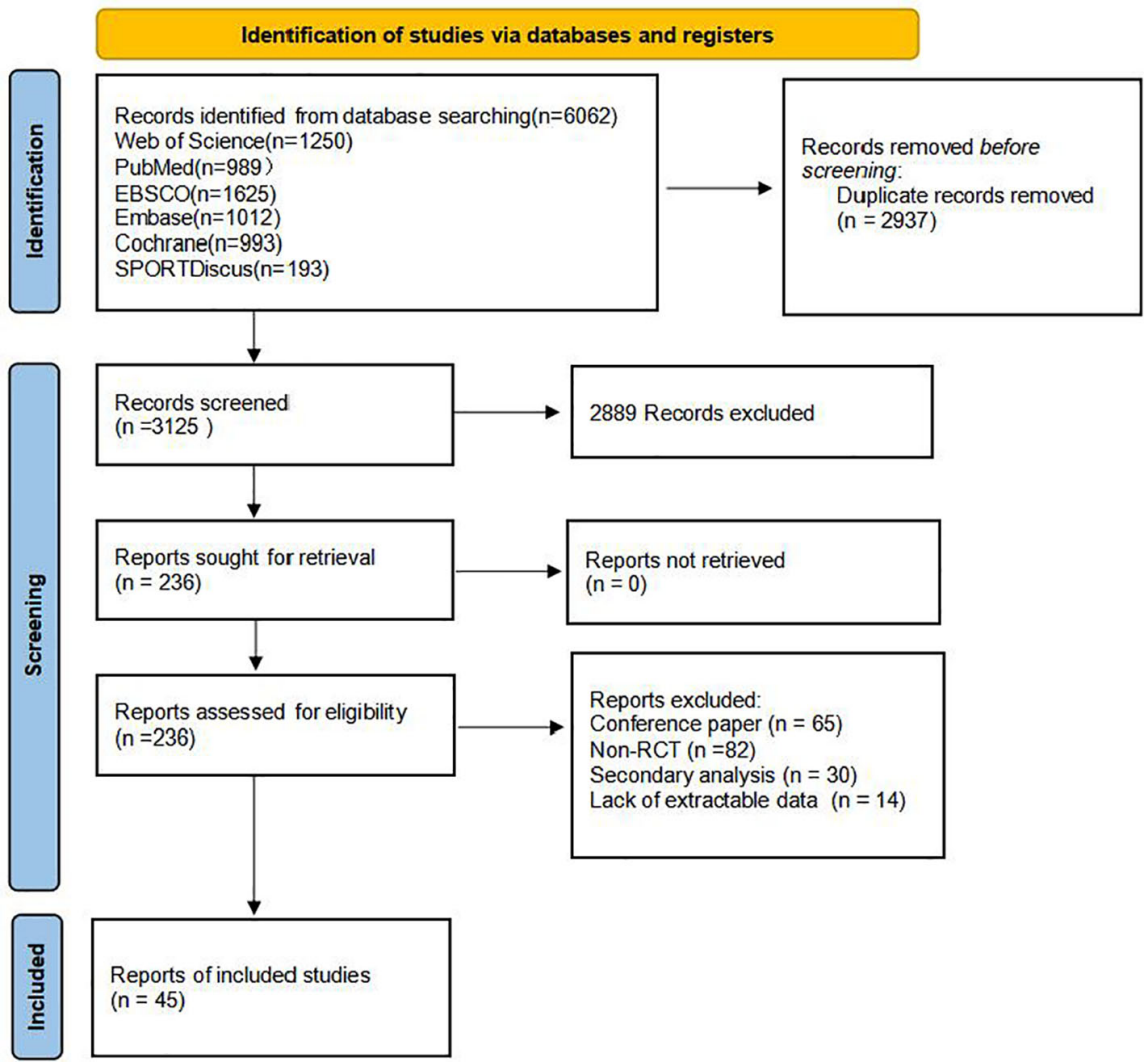
PRISMA flow diagram of the study process.

The study initially retrieved 8062 records from multiple data sources. After removing duplicate records, 5125 records were screened based on titles and abstracts, and 262 full-text articles were obtained to assess their eligibility for inclusion. In the end, 45 studies were included, involving a total of 2358 children and adolescents (See **Table 3**), These can be categorised into three classic paradigms: response inhibition tasks, comprising 18 studies on the Stop-Signal task and 15 studies on the Go/No-Go task; and interference inhibition tasks, comprising 8 studies on the Stroop colour-word task and 4 studies on the Flanker task. The effect sizes were uniformly converted into standardised mean differences (SMD). See **Figure 1** for details of the screening process.

**Table 3:** Basic characteristics of included studies.

Author & tear	Country	Sample size		Mean age (years)		Instrument	Type
		E	C	E	C		
Verret et al,2012 (24)	Canada	10	11	9.10 ± 1.1 0		Combined exercise	ADHD
Ziereis et al,2015 (25)	Germany	13/14	16	9.20 ± 1.30	9.5 ± 1.40	Aerobic exercise/Sports games	ADHD
Ji et al,2022 (26)	South Korea	16	14	10.50 ± 1.20		Combined exercise	ADHD
Kadri et al,2019 (27)	Tunisia	20	20	14.50 ± 3.50	14.20 ± 3.00	Martial arts sports	ADHD
Memarmoghaddam et al,2016 (28)	Iran	19	17	8.31 ± 1.29	8.29 ± 1.31	Combined exercise	ADHD
Hoza et al,2015 (29)	United States	94	108	6.83 ± 0.96		Aerobic exercise	ADHD
Bustamante et al,2016 (30)	United States	19	16	9.40 ± 2.20	8.70 ± 2.00	Sports games	ADHD
Choi et al,2015 (31)	South Korea	13	17	15.80 ± 1.70	16.0 0± 1.20	Aerobic exercise	ADHD
Chou et al,2017 (32)	China	25	25	10.71 ± 1.00	10.30 ± 1.07	Mind–body exercises	ADHD
Benzing et al,2019 (33)	Switzerland	28	23	10.46 ± 1.30	10.39 ± 1.44	Combined exercise	ADHD
Liang et al,2022 (34)	China	40	40	8.37 ± 1.42	8.29 ± 1.27	Combined exercise	ADHD
Jensen et al,2004 (35)	Australia	11	8	10.63 ± 1.78	9.35 ± 1.70	Mind–body exercises	ADHD
Nejati et al,2021 (36)	Iran	15	15	9.43 ± 1.43		Combined exercise	ADHD
Pan et al,2016 (37)	China	16	16	8.93 ± 1.49	8.87 ± 1.56	Ball sports	ADHD
Silva et al,2020 (38)	Brazil	18	15	12.00 ± 2.00	12.00 ± 1.00	Aerobic exercise	ADHD
Berg et al,2019 (39)	Netherlands	263	249	10.50± 1.30		Combined exercise	ADHD
Hattabi et al,2019 (40)	Tunisia	20	20	9.95 ± 1.31	9.75 ± 1.33	Aerobic exercise	ADHD
Rezaei et al,2018 (41)	Iran	7	7	9.10 ± 1.30		Mind–body exercises	ADHD
Chang et al,2022 (42)	China	16	16	8.31 ± 1.30	8.38 ± 1.20	Ball sports	ADHD
Li et al,2025 (43)	China	60	60	8.40 ± 1.30		Mind–body exercises	ADHD
Afshari et al,2012 (44)	Iran	20	20	9.1 ± 1.8	9.0 ± 1.7	Combined exercise	ASD
Borgi et al,2016 (45)	Italy	15	13	9.2 ± 1.8	8.0 ± 1.5	Aerobic exercise	ASD
Chan et al,2013 (46)	China	20	20	11.28 ± 3.90	12.42 ± 3.25	mind-body exercises/Aerobic exercise	ASD
Tse et al,2019 (47)	China	19	21	10.11 ± 1.20	9.81 ± 1.17	Ball sports	ASD
Phung et al,2019 (48)	United States	14	20	9.10 ± 1.10	9.52 ± 1.07	Martial arts sports	ASD
Pan et al,2017 (49)	United States	11	11	9.68 ± 1.61	8.49 ± 1.76	Ball sports	ASD
Yang et al,2021 (50)	United States	15	15	4.67 ± 0.70	5.03 ± 0.55	Ball sports	ASD
Wang et al,2020 (51)	South Korea	18	15	5.11 ± 0.65	4.70 ± 0.70	Ball sports	ASD
Milajerdi et al,2021 (52)	Iran	20	20	7.95 ± 1.60	8.45 ± 1.43	Sports games	ASD
Nekar et al,2022 (53)	South Korea	12	12	14.42 ± 5.14	14.17 ± 5.09	Combined Exercise	ASD
Greco et al,2020 (54)	Italy	14	14	9.07 ± 1.00	9.43 ± 1.02	Martial arts sports	ASD
Koenig et al,2012 (55)	United States	24	22	9.58 ± 1.17	8.58 ± 1.17	mind-body exercises	ASD
Su et al,2025 (56)	United States	21	19	8.2 ± 0.4	9.0 ± 0.6	Combined exercise/Aerobic exercise	ASD
Ji et al,2022 (57)	China	34/33	33	12.5 ± 2.36	12.8 ± 2.69	Combined exercise/Ball sports	ASD
Zhang et al,2025 (58)	China	15	13	4.90 ± 0.66	4.77 ± 0.70	Ball sports	ASD
Bremer et al,2020 (59)	Canada	12	12	11.1 ± 1.3		Aerobic exercise	ASD
Zhao et al,2024 (60)	China	16	14	10.69 ± 0.94	10.79 ± 1.05	Aerobic exercise	ASD
Saad et al,2024 (61)	Egypt	16	16	6		Combined exercise	ED
Piedra et al,2024 (62)	Ecuador	15	15	12.73 ± 1.43	12.60 ± 1.30	Sports games	MID
Chen et al,2024 (63)	China	15	15	5.27 ± 0.70	5.07 ± 1.10	Sports games	ASD
Zhang et al,2025 (64)	China	8	8	3.00 ± 0.34		Sports games/Combined exercise	ASD
Chen et al,2015 (65)	China	45	41	10.6 ± 3.6	10.7 ± 4.0	Ball sports	MID
Emami et al,2019 (66)	Iran	15	15	8.7 ± 0.6	8.6 ± 0.8	Combined exercise	LD
Tsai et al,2009 (67)	China	13	14	9.53 ± 0.36	9.47 ± 0.29	Ball sports	DCD
Damanpak et al,2022 (68)	Iran	15	15	10.8 ± 0.4	10.6 ± 0.5	Sports games	DCD

E, Experimental Group; C, Control Group.

### 3.2 Introduction to the basic characteristics of the included studies

Among the 45 included studies, the types of exercise interventions in the experimental group primarily included combined exercises, combat sports, ball games, sports games, coordination training, mind-body exercises, single aerobic exercises, and cycling exercises. The average age of participants was 9.0 ± 2.6.Most of the literature reviews focus on ADHD and ASD, comprising a total of 39 studies, whilst only six studies cover DCD, MID, ED and LD. Combined exercises refer to exercises that combine two or more different movement patterns in a single training session. Ball sports are exercises that use a ball as the central equipment. Sports games are gamified exercises that use physical activity as a medium, rules as a foundation, and entertainment and education as primary objectives. Coordination training involves specialized exercises targeting the coordinated functioning of the nervous and muscular systems. Mind-body exercises emphasize the integration of awareness, breathing, and movement to improve physical function. Aerobic exercise refers to sustained low-intensity repetition of the same movement. Cycling involves primarily seated riding movements. Detailed inclusion criteria for the literature are presented in **Table 1**.

### 3.3 Relevant content on quality assessment of included literature

**Figure 2:**
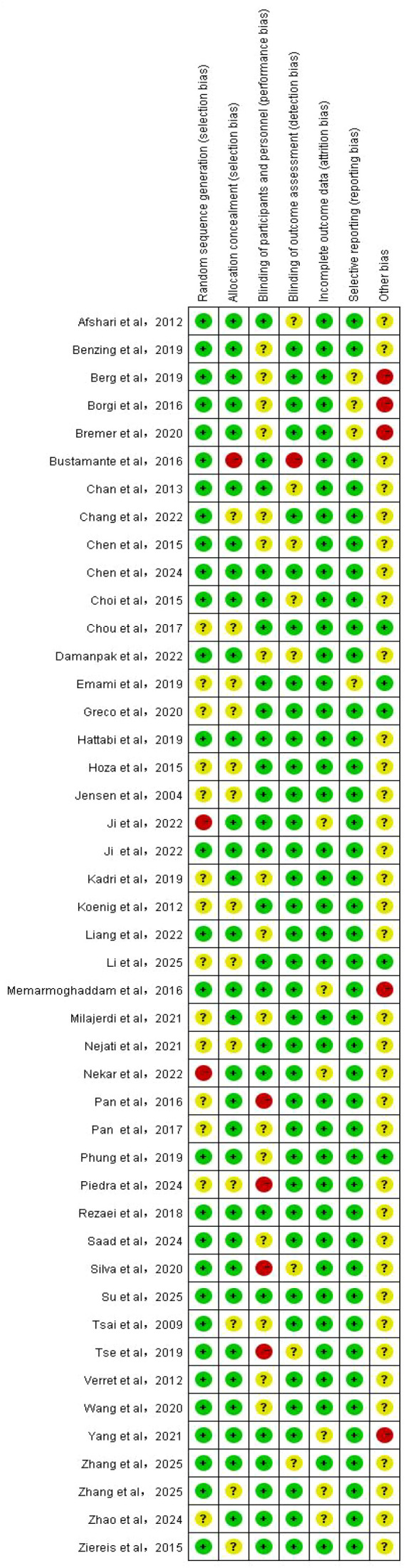
Bias risk diagram for each item.

**Figure 3:**
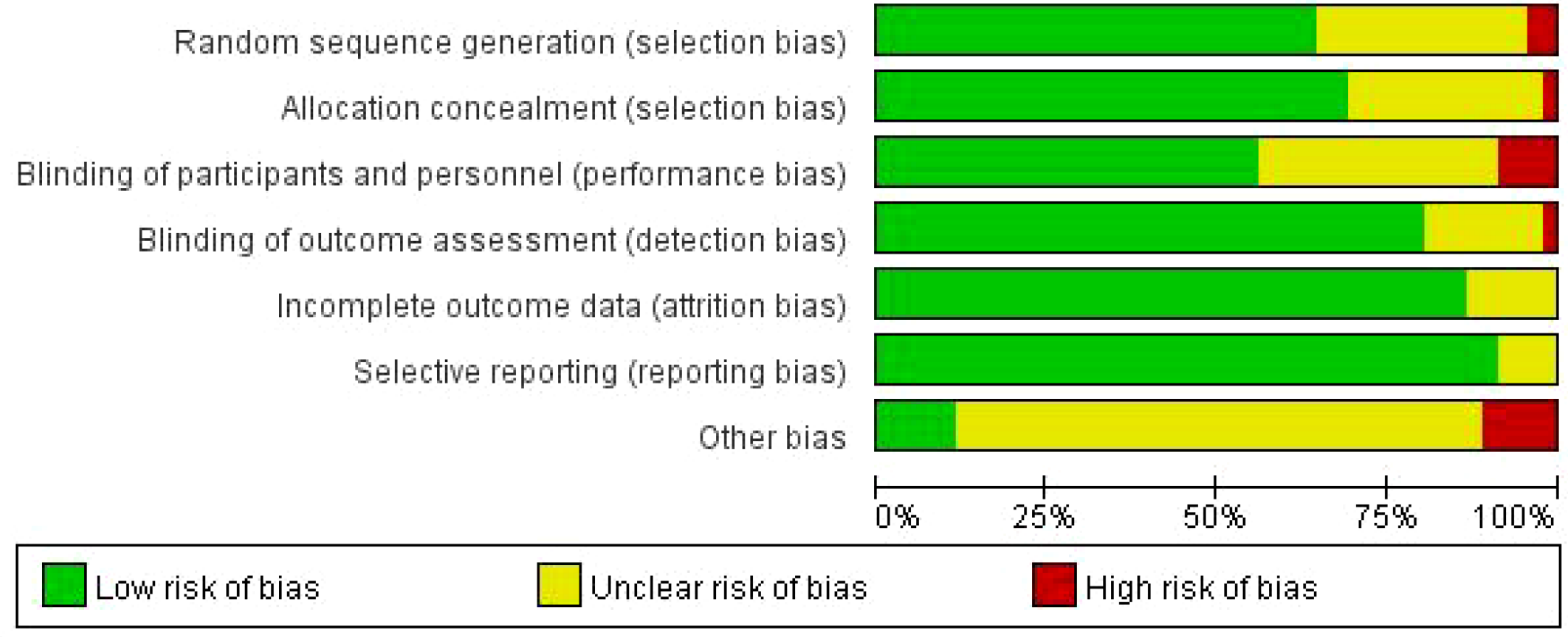
Overall bias risk diagram.

The RevMan 5.4 software was used to conduct the bias risk assessment and systematic quality evaluation of the 45 included randomized controlled trials (69). **Figure 2** presents the bias risk assessment results of these 45 studies across seven domains: ① random sequence generation, ② allocation concealment, ③ blinding of participants, ④ blinding of result assessors, ⑤ integrity of data, ⑥ selective reporting, ⑦ other potential biases. **Figure 3** presents a three-color bar graph (green represents low risk, yellow represents some concerns (unclear risk), and red represents high risk) to summarize the overall distribution of bias risks in each dimension. Given that various types of exercise interventions were included, and blinding of participants and personnel was difficult to maintain, the high risk of bias in most studies was attributed to the inability to maintain blinding.

### 3.4 Network meta analysis results

#### 3.4.1 Network diagram

**Figure 4:**
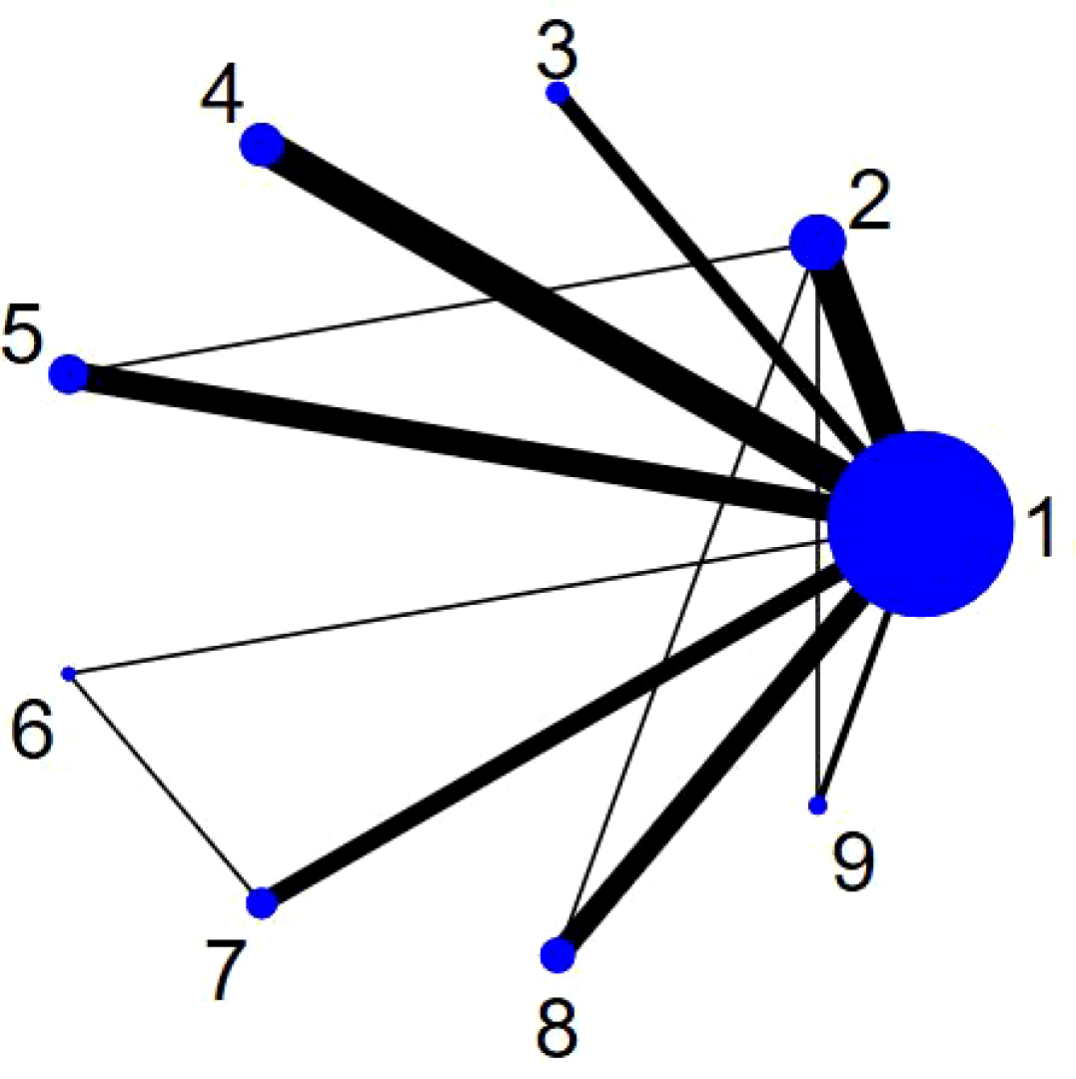
Network evidence diagram. 1, con; 2, combined exercise; 3, martial arts sports; 4, ball sports; 5, sports games; 6, coordination exercises; 7, mind–body exercises; 8, aerobic exercise; 9, cycling.

**Figure 4** shows the network structure of the intervention measures in this network meta-analysis. Each circular node in the figure represents an intervention measure, and the node area is in direct proportion to the study sample size corresponding to the measure. The larger the circle means the more studies included in the intervention measure. The thickness of the lines is proportional to the amount of evidence from the comparisons; thicker lines indicate more studies and more robust evidence. Through this network diagram, one can intuitively assess the relative positions of each intervention within the network, the distribution of sample sizes, and the density of evidence, providing a structural basis for subsequent effect estimation and ranking.

#### 3.4.2 Comparison results between the constituent elements of the exercise type

**Figure 5:**
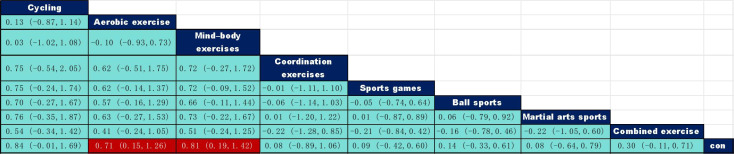
League table of pairwise comparisons of intervention effects among exercise type elements. The red numbers are statistically significant.

A network meta-analysis framework was employed to present the results of pairwise comparisons between different types of exercise using a league table. As shown in **Figure 5**, the standardized mean difference (SMD) and 95% confidence interval are presented below the diagonal line of the league table, whereas the upper and lower limits of the confidence interval are presented above the diagonal line. red markings indicate statistical significance (P < 0.05). Based on direct evidence from 45 RCTs, both single-modality aerobic exercise (SMD = 0.71, 95% CI: 0.15–1.26) and mind-body exercise (SMD = 0.81, 95% CI: 0.19–1.42) were significantly superior to conventional control groups; other exercise types (cycling, coordination training, ball sports, martial arts and combined exercise) did not reach statistical significance when compared with control groups. The above results are based on a unified set of inhibitory control outcome measures, encompassing behavioural assessments such as the Stop-Signal Task, Go/No-Go Task and Stroop Task; all measures were converted to SMD for cross-study comparison.

Notably, a direct comparison between mind-body exercise and single-modality aerobic exercise showed no significant difference (SMD = 0.10, 95% CI: −0.45–0.65). however, the SUCRA probability ranking indicated a higher likelihood that mind-body exercise was the optimal intervention (87.1% vs 80.5%). Some comparisons in the league table lack direct evidence, and their effect estimates rely on indirect information; therefore, interpretation should be undertaken with caution. In summary, the current evidence supports both single-modality aerobic exercise and mind-body exercise as effective intervention options for improving inhibitory control in children and adolescents with neurodevelopmental disorders, with mind-body exercise potentially offering superior relative efficacy.

#### 3.4.3 Probable ranking of the best interventions for each exercise type

**Figure 6:**
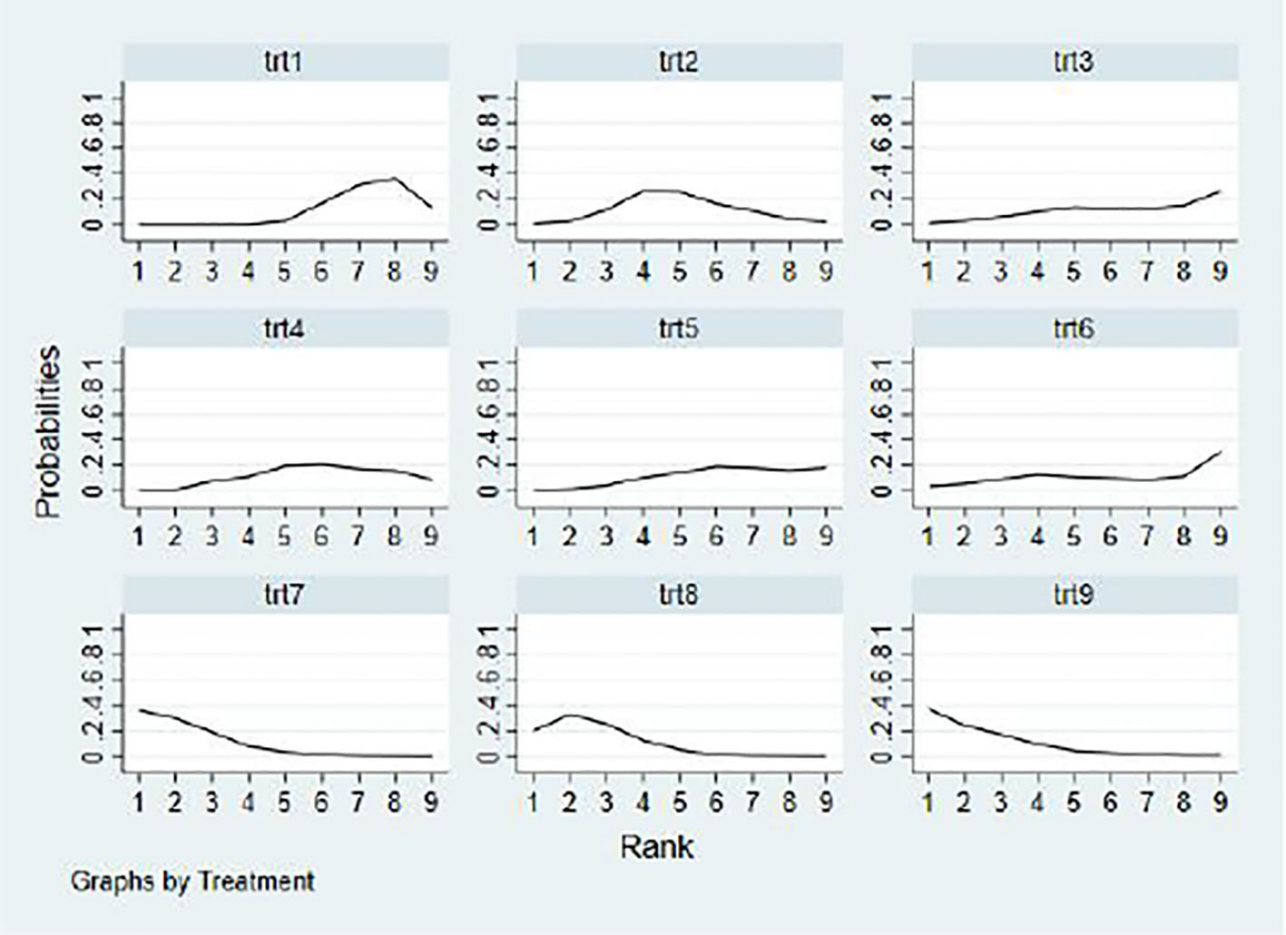
Probability ranking of dose–response effects for exercise type components. 1,con; 2, combined exercise; 3, martial arts sports; 4, ball sports; 5, sports games; 6, coordination exercises; 7, mind–body exercises; 8, aerobic exercise; 9, cycling.

Based on the SUCRA values in **Table 4** and **Figure 6**, the effectiveness of different types of exercise intervention in children and adolescents with neurodevelopmental disorders was ranked as follows, mind-body exercises (SUCRA = 87.1) > cycling (SUCRA = 85.2) > aerobic exercise (SUCRA = 80.5) > combined exercises (SUCRA = 69.8) > ball sports (SUCRA = 52.1) > coordination training (SUCRA = 43.6) > martial arts (SUCRA = 34.1) > sports games (SUCRA = 31.5) > control group (SUCRA = 20.1).

**Table 4:** SUCRA values for assessing the efficacy of intervention components across different exercise types.

Rank	Type	SUCRA	PrBest	MeanRank
1	Mind–body exercises	87.1	36.5	2.0
2	Cycling	85.2	37.5	2.2
3	Aerobic exercise	80.5	20.4	2.6
4	Combined Exercise	69.8	0.7	3.4
5	Ball sports	52.1	0.3	4.8
6	Coordination exercises	43.6	3.2	5.5
7	Martial arts sports	34.1	1.1	6.3
8	Sports games	31.5	0.3	6.5
9	Con	20.1	0.0	7.4

### 3.5 Sensitivity analysis

The sensitivity analysis in this study was conducted using the leave-one-out method. After sequentially removing each included study, the pooled effect size fluctuated between 0.650 and 0.856. This result indicates that the impact of any single study on the overall conclusion is relatively minor, suggesting that the results are highly robust.

### 3.6 Publication bias test

**Figure 7:**
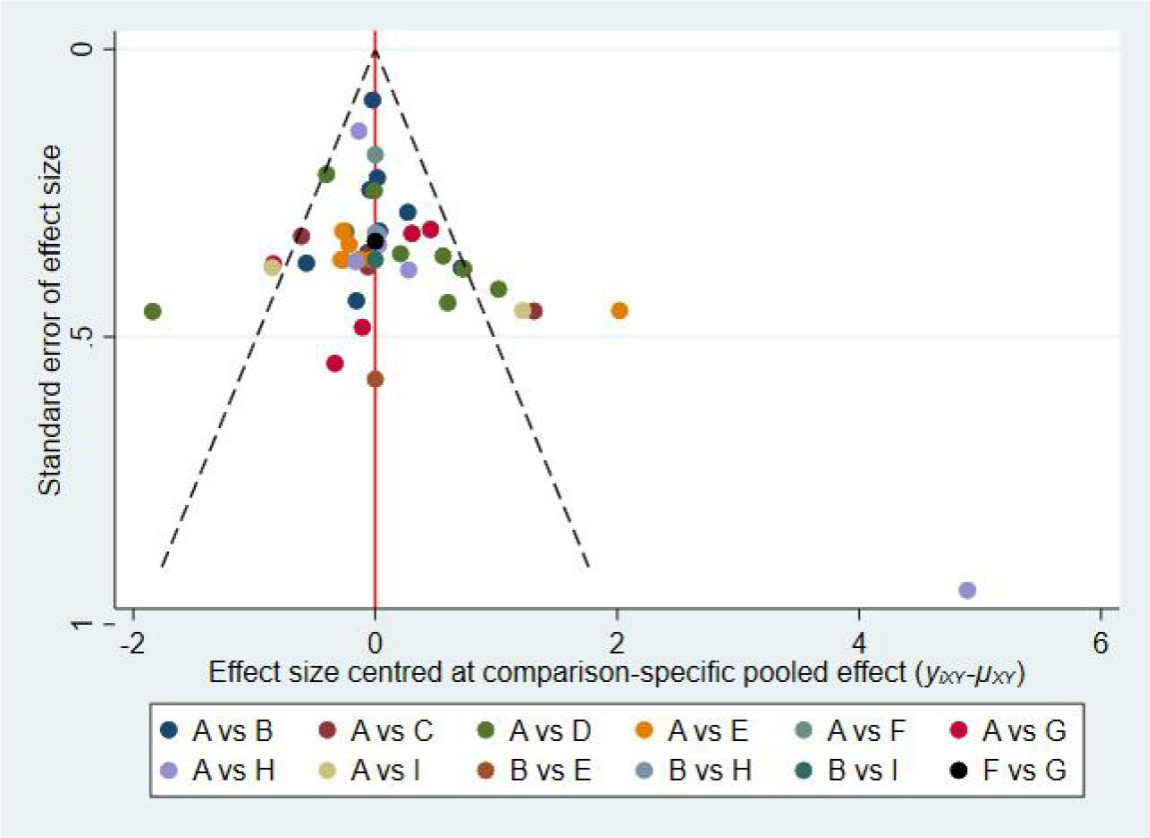
Funnel plot of dose-response effects of exercise prescription elements. 1,con; 2, combined exercise; 3, martial arts sports; 4, ball sports; 5, sports games; 6, coordination exercises; 7, mind–body exercises; 8, aerobic exercise; 9, cycling.

The results of the publication bias assessment are shown in **Figure 7**. The funnel plot indicates that most studies were clustered at the top of the funnel and were symmetrical on both sides, with only a few located outside the funnel, suggesting a low likelihood of publication bias. Further quantitative testing was conducted using Egger’s linear regression method, yielding Egger = 0.076 (p > 0.05), which did not reach statistical significance, indicating no obvious publication bias. However, given the limited number of included studies, caution should still be exercised when interpreting the results.

## 4 Discussion

This study conducted a network meta-analysis of 45 included studies to compare the inhibitory control effects of eight different types of exercise on neurodevelopmental disorders in children and adolescents. The results showed that mind-body exercise (SUCRA = 87.1) ranked first, outperforming combined exercise, ball games, sports games, coordination training, single aerobic exercise, and cycling; with effect sizes significantly higher than the control group (SMD = 0.81, 95% CI: 0.19–1.42). Both ranking and effect size evidence suggest that mind-body exercise may be the optimal exercise type for improving inhibitory control in this population. This conclusion aligns with previous meta-analyses, Previous meta-analyses of chronic exercise interventions for this population have shown that chronic exercise can significantly improve core inhibitory control deficits in children. Specifically, for children with ADHD/ASD, any chronic exercise programme that meets the criteria of regular frequency and combines cognitive and motor coordination components can effectively improve their core inhibitory control. Among these, ball sports, martial arts, motion-based games, and orienteering were identified in this review as high-frequency, effective types for improving inhibitory control (21, 70). This study further clarifies that mind-body exercise interventions may be the most effective. Additionally, the results of this study differ from previous network meta-analyses targeting single NDDs. Previous studies have shown that taekwondo has the best intervention effect on inhibitory control in ADHD (71), and ball sports are optimal for inhibitory control in ASD (72), while this study found that mind-body exercise is more effective. The primary reason for these differences may be the distinction between single symptoms and comorbid symptoms. Research has shown that NDDs rarely occur in isolation and are often accompanied by comorbidities or cross-symptomatic manifestations (9). This study included all comorbid symptoms in the analysis, resulting in outcomes that differ from previous single-condition studies. Additionally, future research should consider how individual factors such as patient gender, age, and medication status interact to produce new effects when comorbidities are present, thereby further validating the optimal intervention type.

The reason why mind-body exercises have become the optimal form of exercise intervention may be related to the characteristics of such exercises, which primarily include qigong, tai chi, and yoga. These exercises are characterized by their slow pace, allowing practitioners to synchronize their breathing with each slow movement to deeply experience the subtle changes in their bodies (73).

From the perspective of cortical inhibitory mechanisms, deficits in inhibitory control in adolescents with neurodevelopmental disorders (NDDs) manifest not only as behavioural impulsivity but also, at a deeper level, stem from a reduction in GABAergic intracortical inhibitory function. As key inhibitory interneurons responsible for maintaining the cortical excitation-inhibition balance, the dysfunction of parvalbumin-positive (PV+) interneurons leads to excessive excitation of pyramidal neurons in the prefrontal cortex and a decline in the efficiency of inhibitory control signal transmission (74). The rhythmic, slow movements of mind-body exercises, combined with deep, rhythmic abdominal breathing, may restore inhibitory control function in the prefrontal cortex by activating the vagus nerve–thalamus–cortex pathway, upregulating GABA_A receptor-mediated synaptic inhibition, and enhancing the activity of PV+ interneurons (75). Mind-body exercises emphasise intention over force, focusing attention on specific body parts; in practice, under conditions of low peripheral load, they enhance inhibitory control over the corticospinal tract via the prefrontal-basal ganglia-thalamic circuit, thereby improving the efficiency of inhibiting irrelevant motor impulses (76). This type of exercise emphasises the trinity of movement, breathing, and intention. Through slow, symmetrical, and repeatable postural transitions combined with deep, rhythmic abdominal breathing (77), it synchronously regulates the balance between the vagus and sympathetic nervous systems, reduces stress hormone levels, and provides a stable environment for the prefrontal network (78). Furthermore, the complexity of these exercises is controllable; the range of motion and balance requirements can be gradually increased to suit the individual characteristics of participants. This approach avoids the sensory overload that high-impact exercise may induce, whilst continuously providing a moderate challenge that promotes neuroplasticity (79). Finally, mind-body exercises are typically practiced and meditated upon in group settings to enhance self-control, reduce anxiety, and decrease impulsive behavior, thereby indirectly improving inhibitory control. In summary, mind-body exercises, with their combined characteristics of “chronic, low-intensity, high-focus, and emotionally friendly, ” offer children with neurodevelopmental disorders a practical and sustainable exercise method, potentially outperforming other exercise types in improving inhibitory control.

Neurobiological studies have shown that the slow movements involved in such exercises can regulate local perfusion pressure by increasing the average blood flow velocity in the posterior cerebral artery (by 22.2%) while decreasing blood flow velocity in the middle cerebral artery (by 23.1%). These differential hemodynamic changes may enhance metabolic demand in the prefrontal-striatal circuit, thereby promoting synaptic plasticity in related synapses. Concurrently, it increases prefrontal cortex thickness, improves white matter integrity, and enhances the efficiency of detecting and inhibiting conflict signals. Chronic, rhythmic mind-body exercises may stabilize these structures, whereas high-intensity intermittent stimuli from combat and ball sports may not achieve the same effects (80). Other studies have shown that mindfulness-based movement synergistically activates the prefrontal cortex, anterior cingulate cortex, and insula-amygdala circuits. fMRI findings indicate that in the “distraction-shift-focus” chain, the anterior cingulate cortex responds first and regulates the sympathetic locus coeruleus norepinephrine system to dynamically optimize environmental adaptation, thereby potentially enhancing response inhibition (81). In contrast, while combined exercises simultaneously activate the motor cortex and prefrontal cortex (82), they lack systematic training combining breathing and attention, and thus may be less effective in terms of effect size compared to mind-body exercises. Meanwhile, while the high situational uncertainty of ball sports promotes cognitive flexibility (83), they may have limited specificity in stimulating inhibitory control.

## 5 Limitations

First, the literature included primarily focused on exercise itself and did not strictly control for confounding factors such as age, gender, diet, baseline physical fitness, and concomitant medication use. Second, although the funnel plot is generally symmetrical and the Egger test yields a p-value >0.05, publication bias cannot be completely ruled out, especially since most of the included studies are small-scale positive results; Furthermore, the existing evidence is heavily concentrated on children with ADHD and ASD, whilst evidence for other subtypes of neurodevelopmental disorders is scarce; there may be differences between subtypes in terms of patterns of inhibitory control deficits and responsiveness to motor interventions; finally, the suitability of certain types of exercise for younger children remains unclear.

## 6 Implications for research

The findings of this study provide a solid theoretical basis for improving inhibitory control in children and adolescents with neurodevelopmental disorders through exercise intervention: the network meta-analysis identifies mind-body exercise as the optimal approach for improving inhibitory control and reveals the core characteristics of “chronic, low-intensity, high-focus” intervention, which can be translated into prescription templates for clinical and school settings.

## 7 Clinical practical significance

This study demonstrates that evidence-based sequencing of mind-body exercises provides a basis for physicians to prescribe exercise regimens and design exercise programs tailored to individual patients. Schools can incorporate mind-body exercises into their curricula, with physical education teachers implementing exercise interventions for children and adolescents with this condition. More importantly, exercise interventions can be used in conjunction with central nervous system stimulants and medications such as atomoxetine to gradually reduce dependence on drugs, opening up new perspectives for comprehensive treatment.

## 8 Conclusion

A total of 45 randomized controlled trials were integrated, and a network meta-analysis was conducted to compare the effects of different exercise types on inhibitory control in children and adolescents with neurodevelopmental disorders. The results suggest that mind-body exercise may be the optimal choice to improve inhibitory control, providing an evidence-based basis for selecting appropriate exercise programs in clinical practice and family education. However, given the limited number of existing studies, the conclusions should be interpreted with caution. Future research should expand the scale of research and include randomized controlled trials involving different types of neurodevelopmental disorders, as well as varying ages and genders, to further enhance the reliability of the results.

## Data Availability

The original contributions presented in the study are included in the article/supplementary material. Further inquiries can be directed to the corresponding author.
